# Clinico-Hematological Findings of Acute Pediatric Visceral Leishmaniasis Referred to the Northeast of Iran during 2005–2015

**Published:** 2020

**Authors:** Shaghik BARANI, Habibollah TURKI, Reza SHAFIEI, Fatemeh JAFARZADEH, Hoda HOSSEINZADEH MALEKI, Saber RAEGHI

**Affiliations:** 1. Department of Immunology, School of Medicine, Shiraz University of Medical Sciences, Shiraz, Iran; 2. Infectious and Tropical Diseases Research Center, Hormozgan Health Institute, Hormozgan University of Medical Sciences, Bandar Abbas, Iran; 3. Vector-Borne Diseases Research Center, North Khorasan University of Medical Sciences, Bojnurd, Iran; 4. Department of Parasitology, Shahid Beheshti Universtiy of Medical Sciences, Tehran, Iran; 5. Mashhad University of Medical Sciences, Mashhad, Iran; 6. Department of Laboratory Sciences, Maragheh University of Medical Sciences, Maragheh, Iran

**Keywords:** Visceral leishmaniasis, *Leishmania infantum*, Bone marrow examination, Hematological features

## Abstract

**Background:**

To characterize the epidemiological, clinical, hematological and biochemical features of 33 cases hospitalized with pediatric visceral leishmaniasis (PVL) in North Khorasan Province of Iran from 2005 to 2015.

**Methods:**

The serological, hematological and biochemical tests were employed in 33 children between 8 months to 6 yr with a final diagnosis of acute visceral leishmaniasis (VL). The diagnosis of VL was established by microscopic demonstration of *Leishmania* spp. amastigotes inactive bone marrow aspiration (BMA).

**Results:**

The most common presenting features were anemia (82.5%), fever (75%), and hepatosplenomegaly (45.4%). Various hematological parameters showed that most patients were suffering from moderate to severe microcytic hypochromic anemia (78.8% had RBC count less than 4 million cells/ul, 67.7% Hb less than 8 fl). 66.7% of them were leukopenic (WBC: less than 5× 10**^3^**/μL) and 24.2% had decreased platelet counts. Pancytopenia was observed in 18.2% of cases. MCV, MCH, and MCHC levels were below the reference range in 88%, 90% and 85.1% of the patients respectively. Moreover, aspartate transaminase (AST) and alanine transaminase (ALT) levels were increased in 53.33% and 6.66% of the patients respectively. 92.9% of cases were C-reactive protein (CRP) positive. Bone marrow was found hyper-cellular in all of them, and myeloid to erythroid ratio (M/E) was more than 4 in 39.1% of cases. Plasma cells slightly were increased in 60% of patients and megakaryocytes were decreased in thrombocytopenic patients.

**Conclusion:**

Bone marrow/splenic aspiration still remains the gold standard test despite its risk and pain for patients.

## Introduction

Zoonotic visceral leishmaniasis (VL) also known as kala-azar or black fever as a main public health problem in the Mediterranean countries is a disseminated parasitic disease transmitted by female Phlebotomine sandflies and is caused by the *Leishmania infantum* (1).

The disease describes a varied spectrum of clinical manifestations in humans, especially in children up to 12 yr old and also in immunocompromised and immunosuppressed adult individuals, such as HIV- patients (2–6). This infection beside some parasitic diseases are endemic in Iran (7–10).

Approximately 500,000 new cases of VL annually have been reported from 98 countries in the world (3–5). The major foci of disease in Iran are Meshkin-Shahr District, Ardabil Province, some areas of Jahrom district in Fars Province and in the North Khorasan province but the disease has been reported sporadically from other regions of the country, too (5, 11–14). Since various clinical and hematological manifestations are found in VL (15), most of physician may misdiagnose the disease. Delay in diagnosis can lead to failure in the treatment which results in death in most cases with PVL (16, 17). Demonstration of the amastigote forms of the parasite in aspirate samples of bone marrow or spleen is made confirm of diagnosis (12, 18).

The aim of the present study was to characterize the epidemiological, clinical, hematological and biochemical features of 33 cases hospitalized with PVL in North Khorasan Province of Iran.

## Methods

### Population study

This study was conducted at hospitals affiliated with the North Khorasan University of Medical Sciences, Bojnurd, Iran. The epidemiological and clinico-hematological features of 33 children between 8 months to 6 yr with a final diagnosis of acute visceral leishmaniasis (VL) were reviewed from 2005 to 2015. The diagnosis of VL was established by microscopic demonstration of *Leishmania* spp. amastigotes inactive bone marrow aspiration (BMA) and was examined for cellularity.

Demographic and clinical information including age, sex, signs, and symptoms of persistent systemic infection such as duration of disease, fatigue, loss of appetite, weight loss, physical examination findings and treatment were recorded. In addition, sonography and radiologic findings, laboratory records including hematological, serological and biochemical analysis of blood and urine, were reviewed.

Informed consent was obtained from parents of all children participants included in the study. Furthermore, ethical approval for the research (IR.nkums.REC.1396.49) was obtained by the ethics committee of North Khorasan University of Medical Sciences.

### DAT Test

Sera from the fresh whole blood was used in direct agglutination test (DAT) for detection of anti-*Leishmania* antibodies. The DAT antigen was obtained from Medical Parasitology and Mycology Department at Tehran University of Medical Sciences. DAT was performed, according to a protocol previously described in “Visceral leishmaniasis in Iran: review of the epidemiological and clinical features” in Booyerahmad district (19).

### Data analysis

Data were collected on a data sheet and analyzed by SPSS ver. 16 (Chicago, IL, USA). Chi-square and Fisher's exact test was used to determine the association of VL disease and demographic or clinical-hematological features by comparing the proportions of any variable in different groups. The descriptive analysis was made through the distribution of frequency, mean and confidence interval of 95% (95% CI), according to the variable type. The *P*-value less than 0.05 was considered statistically significant.

## Results

### Epidemiological features of the VL patients

Age distribution of patients with visceral leishmaniasis was 7 months to 6 yr old. The majority of the cases (60%) was between 2–3 yr of age and 18% were younger than 1. Mean±SD and median age of the patients was 2.65±1.57 and 2.5 yr respectively. From 33 cases of VL, 23 (69.7%) were female and 10 (30.3%) were male, with a female: male ratio of 2.3:1. Cases were from different peripheral districts in the North Khorasan Province. About 80% were from rural areas or were nomads and most cases (70%) were from Bojnurd, Raz, and Jargalan areas.

**Fig. 1:**
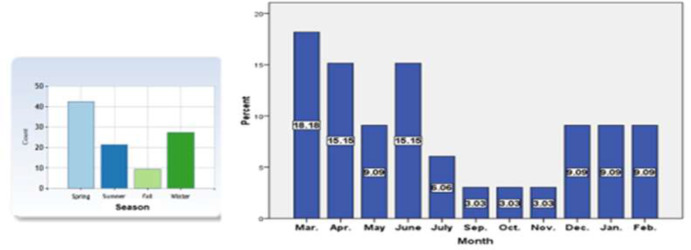
Pediatric visceral leishmaniasis distribution throughout the year

To investigate the climate change impact on the prevalence of PVL, we classified our patients into 4 seasonal groups based on hospital admission date (Fig. 1). The highest incidence of disease was in the spring (42.4%), then winter (27.3%), summer (21.2%), and the lowest incidence in the fall (9.1%). The most prevalence of PVL was in Mar (18.2%), then Apr (15.2%) and Jun (15.2%). Reducing the number of admitted patients in Sep (3%), Oct (3%), Nov (3%), and no patient were admitted in Aug.

### Clinical features of the VL patients

**Fig. 2:**
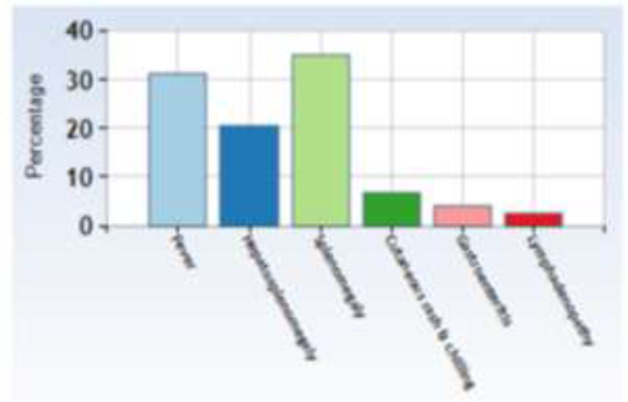
Pediatric visceral leishmaniasis clinical features

Each patient with VL has certain clinical signs and symptoms (Fig. 2). Anemia (33 cases: 82.5%), fever (28 cases: 75%) and hepatosplenomegaly (21 cases: 52.5%) were the commonest presenting feature of the disease. Splenomegaly (without hepatomegaly) was seen in 26 patients (78.8%). Other noticeable clinical features of the patients were cutaneous rash and chilling (5 cases: 15.15%), gastroenteritis (3 cases: 9.09%), lymphadenopathy (2 cases: 6.06%). Disease duration which considered as the interval between the dates of patient’s admission and discharge was 17.32±7.83 day.

### Hematological features of the VL patients

**Fig. 3:**
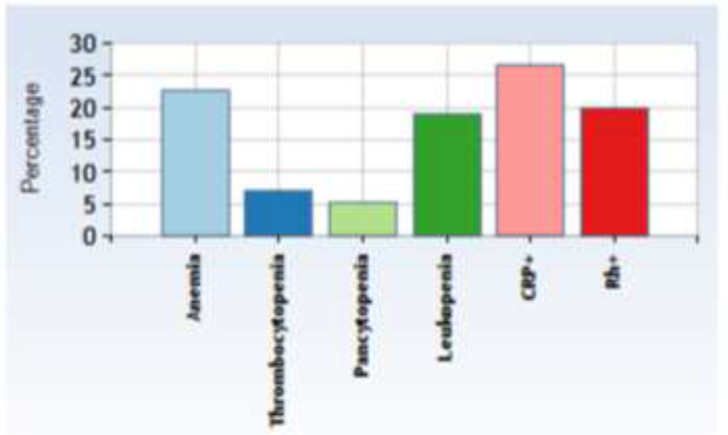
Pediatric visceral leishmaniasis peripheral blood picture

Various hematological parameters including white blood cell count, differential leucocyte count, red cell indices, etc. show that all patients (100%) were suffering from moderate to severe microcytic hypochromic anemia (Hb: 7.67±1.69 g/dl). Hematological features recorded at admittance are shown in Fig. 3 and Table 1.

**Table 1:** Pediatric visceral leishmaniasis (PVL) disease duration and hematological findings

***Descriptive Statistics***					
	***N***	***Minimum***	***Maximum***	***Mean***	***Std. Deviation***
Disease duration (days)	28	5	34	17.32	7.83
RBC (×10^3^/μL)	31	1.91	4.75	3.48	0.68
Hb (g/dl)	31	5.5	12.5	7.67	1.69
RDW (%)	26	12.3	24	18.32	3.02
MCV (fl)	25	61	101.6	73.74	9.22
MCH (pg)	30	13.71	33.3	22.27	3.93
MCHC (g/dl)	27	18.4	33.8	29.21	3.43
ESR (mm/h)	28	32	110	84.86	15.52
Fe (μg/dL)	12	1.4	55	36.30	19.64
Ferritin (ng/mL)	5	163	740	343.80	254.89
TIBC (μg/dL)	7	18	640	314.86	204.68
WBC (×10^3^/μL)	31	1.4	15.9	5.04	3.11
Lymphocyte	30	30.60	88.10	59.46	15.74
Monocyte	30	.30	11.47	6.73	3.038
Neutrophil	30	9.80	68.70	32.28	18.36
Plt (×10^3^/μL)	30	16	379	115.93	71.30
PT (seconds)	11	13	17	14.14	1.48
PT Activity	11	24.4	100	81.51	25.31
INR	11	1	13	2.25	3.57

Twenty-six patients (78.8%) were (leukopenic) WBC: 5.04 ±3.11×10^3^/μL). Platelet counts were recorded in 33 cases and 8 patients (24.2%) were thrombocytopenic (PLT: 115.93±71.3×10^3^/μL). Increased ESR levels were seen in 100% of cases (84.86±15.52).

The prevalence of thrombocytopenia was significantly much lower in RhD-positive phenotype patients than RhD-negative ones (13.6% vs. 71.4%, *P*=0.008, OR=0.1, CI= 0.01 to 0.48).

Microscopic examination of the patient's peripheral blood smear demonstrated variable degrees of anisocytosis, anisochromia, ovalocytosis, crenated cells, anisopoikilocytosis and inadequate platelet (%RDW: 18.32±3.02). Furthermore, neutropenia (32.28 ± 18.36) and lymphocytosis (59.46 ± 15.74) was observed in differential leukocyte count, but we did not observe any change in monocyte number.

Elevated erythrocyte sedimentation rate (ESR) was reported in 100% of cases 84.86±15.52 and CRP was positive in 92.9% of them.

The measurement of Hb is generally considered accurate than Hct or RBC count for the diagnosis of anemia. Hb is measured directly, whereas Hct in some automated instruments is measured indirectly, calculated from the product of the RBC count times the mean corpuscular volume (MCV). Thus, factors that spuriously affect the RBC count (such as RBC clumping) and/or the MCV will ultimately affect the Hct measurement.

The Mean±SD for hemoglobin concentration were very lower than the reference range (Table 2) in all three age groups (0.5–1.9 yr old: 7.74 ±1.98, 2–5.9 yr old: 7.75±1.54 and 6–12 yr old: 6.60 ± 1.55).

**Table 2:** Hemoglobin concentration in children with visceral leishmaniasis

***Age Groups (yr)***	***N***	***Minimum***	***Maximum***	***Mean***	***Std. Deviation***
0.5–1.9	12	5.50	12.50	7.74	1.98
2–5.9	17	6.00	12.50	7.75	1.54
6–12	2	5.50	7.70	6.60	1.55

From 33 PVL diagnosed cases confirmed with microscopic demonstration of *Leishmania* spp. amastigotes in bone marrow aspiration, DAT was performed in 8 patients which 4 cases were defined seropositive for anti-*L. infantum* (IgG) with titers of ≥1:3200. These children were aged 0.8, 1.5, 2 and 3 yr old.

Remarkable signs and symptoms in 4 cases with DAT positive (≥1:3200) outcomes included fever (50%), anemia (75%), splenomegaly (50%), hepatosplenomegaly (25%) and no lymphadenopathy.

Short-term duration of disease (6, 8, 12 and 15 d) was demonstrated in seropositive children while these periods were longer (15–34 d) in seronegative cases but there was no statistically significant difference between them.

We did not find any statistically significant association between the presence of anti *Leishmania* antibodies and the clinical manifestations of the disease; however, its look like that splenomegaly as well as pancytopenia and especially leukopenia was more prevalent in seropositive patients rather than seronegative ones.

### Biochemical features of the VL patients

Liver transaminases, serum glutamic-oxaloacetic transaminase (SGOT) and serum glutamic-pyruvic transaminase (SGPT) as part of the liver function test was done and it was found that 68% of patients had elevated serum SGOT (AST) levels while we did not find any alteration in serum SGPT (ALT) levels. Serum electrolytes were examined for investigation of changes in effects of disease on body fluids. Hyperkalemia was observed only in 23.1% of patients (7.2 ± 1.38), while Na (sodium) level was within the normal range (Table 3).

**Table 3:** Liver transaminases and electrolytes serum concentration in children with visceral leishmaniasis

***Variable***	***N***	***Descriptive Statistics***			
		***Minimum***	***Maximum***	***Mean***	***Std. Deviation***
SGOT (AST)	25	13	96	57.64	25.10
SGPT (ALT)	25	7	56	22.32	13.89
Alkaline phosphatase (AlkPhos)	12	122	376	250.92	76.21
Na	12	118	141	131.58	7.65
K	13	3.7	8	5.14	1.35

### Bone marrow aspiration (BMA) of the VL patients

**Fig. 4:**
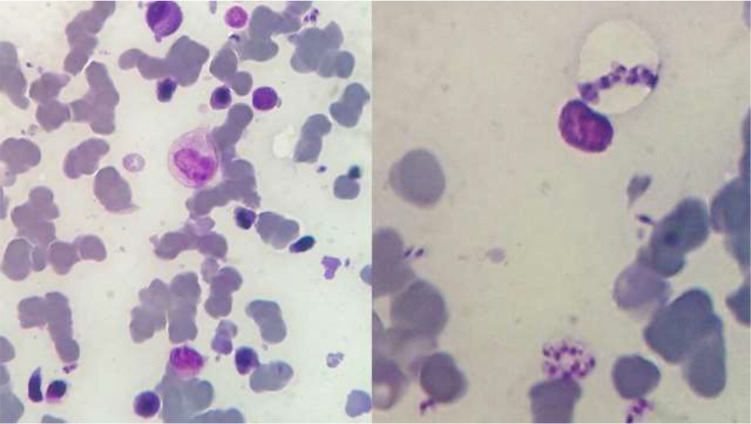
*Leishmania* amastigotes in bone marrow biopsy *Leishmania* amastigotes in bone marrow aspiration specimens from children with visceral leishmaniasis. Note the nucleus and kinetoplast in each amastigote. Reproduced from: Department of Pathobiology and Laboratory Sciences

The mean of myeloid to erythroid ratio (M/E) was 4.57 ± 3.5. Hyper-cellular bone marrow was found in 37.5% of cases with myeloid to erythroid ratio mean (M/E) of 8.11 ±3.25. Plasma cells slightly increased in 60% of cases. Moreover, megakaryocytes were decreased in number in thrombocytopenic patients (Fig. 4).

## Discussion

The results of the current study showed that the highest rate of PVL was occurred during spring, but there were no statistically significant differences in PVL monthly and seasonal distribution of PVL. This study showed obviously that PVL incidence is observed throughout the year, this is likely to indicate the presence of sand flies -the vectors of VL- in all seasons. The highest number of cases was reported in the spring, then winter and lowest number of cases was in the Fall and this is an agreement with Iraqi studies (20, 21) while it is slightly different from a Saudi Arabia study where the peak was in late spring and summer, then fall in winter (22). These observations corresponds to the active season of sand flies in Iran, considering the incubation period between the infected sand fly bite and developing symptoms. The incubation period varies after infection (usually 3–6 month) and may depend on the patient's age and immune status as well as the species of *Leishmania* (23).

In a retrospective clinical-hematological analysis of PVL in southwestern Iran (24), the patients were admitted throughout the year with most cases in Jan (13.9%), Feb (12.4%) and Mar (11.8%) and the least cases in Aug (5%), Sep (5.5%), and Nov (5.5%), while in our current study, the most prevalence of PVL was in Mar (18.2%), then Apr (15.2%) and Jun (15.2%). Reducing the number of admitted patients in Sep. (3%), Oct. (3%), Nov. (3%), and no patient were admitted in August.

Macrophages, as a primary resident cell for *Leishmania* are responsible for the dissemination of parasite to all parts of the reticuloendothelial system, particularly to the spleen, liver, and bone marrow. Splenomegaly with a thickened spleen capsule and significant increase of Billroth reticular cells, packed with amastigote forms of *Leishmania*, was observed in one-third of patients. In the liver, the Kupffer cells are increased in size and number which infected with amastigote forms of *Leishmania*. Macrophages parasitized by *Leishmania* substitute the hematopoietic tissue in the hyperplastic condition of the bone marrow. Pancytopenia as a consequence of hypersplenism was demonstrated in one-fifth of patients (15, 25–26). Based on differences in *Leishmania* virulence strategies, the socioeconomic conditions, immune status, coinfection with other microorganisms and genetic predisposition of individuals infected with parasites, infected patients exhibit a variety of clinical manifestations.

The diagnostic gold standard for VL is the demonstration of amastigotes within the macrophages in the bone marrow aspirates smear (27). The parasite load can be quantified based on a scale from 0 (no parasites in 1000 microscopic fields) to 6+ (>100 parasites per microscopic field) (28).

In VL as a disease of reticuloendothelial system, the proliferation of amastigotes mainly in the mononuclear phagocytic system (MPS) of the spleen, liver and bone marrow result in hepatosplenomegaly and hyperplastic BM. Splenomegaly is more prominent than hepatomegaly (29) which is in line with the findings of current study (78.8% vs. 48.5%). The prevalence of splenomegaly in VL has been found to be as high as 85% to 100% in various studies (29, 30).

Leukopenia as one of the earliest changes in peripheral blood has been observed in about 75% patients with VL in various studies which is slightly more than 66.7% in the current study. Most likely, splenomegaly is considered to be the main cause for leukopenia in these patients (29). Regular increase in erythrocyte sedimentation rate (ESR) in VL is always noted, probably because of release of CRP.

Anemia is common hematological feature of VL but it is more severe in pediatric VL. Accordance with our results, hemoglobin levels mean in many studies were lower than the reference range for children (8.3, 7.8 g/dl) (15) and it falls to a lower Hb level that were reported in other studies (31, 32).

Some studies have reported a protective effect of the RhD-positive phenotype, the most immunogenic D antigen of the Rh blood group system, against the negative effect of latent toxoplasmosis on psychomotor performance in infected subjects (33–35). Since we noticed that the prevalence of thrombocytopenia was significantly much lower in RhD-positive phenotype patients than RhD-negative ones, the Rh factor might also play a protective role. This finding needs further research to confirm the possible protective role of Rh factor in PVL and identify the mechanisms of action.

“Patients of VL generally have hemoglobin levels in the range of 7 to 10 gm/dl” (29) that is in line with our findings. A very high ESR usually had seen in severe infection such as VL (29, 30).

During infection with VL, circulating anti-*Leishmania* antibodies are produced by plasma cells (PCs) against the surface antigens of the invading parasites. The presence of these Abs are associated with duration of disease, but we could not find a statistically significant association between them, it maybe because of low sample size of our seropositive cases. Perhaps one of the reasons is that children under 2–3 yr of age have not the ability to produce antibodies at distinguishable levels due to immaturity of B lymphocytes (Plasma cells), a fact that can be explained the positive DAT tests in only 4 cases with PVL.

## Conclusion

There are a number of invasive and non-invasive tests for diagnosis of VL, but using bone marrow or splenic aspiration is still used as the gold standard test, despite its risk and pain which cause for patients.

## References

[1] Gramiccia M Gradoni L The current status of zoonotic leishmaniases and approaches to disease control. Int J Parasitol. 2005;35(11–12):1169–80. 16162348 10.1016/j.ijpara.2005.07.001

[2] Shafiei R Namdar Ahmadabad H Nezafat Firizi M Cytokine profile and nitric oxide levels in macrophages exposed to *Leishmania infantum* FML. Exp Parasitol. 2019;203:1–7 31128104 10.1016/j.exppara.2019.05.004

[3] Badirzadeh A Heidari-Kharaji M Fallah-Omrani V Antileishmanial activity of Urtica dioica extract against zoonotic cutaneous leishmaniasis. PLoS Negl Trop Dis. 2020; 14(1): e0007843. 31929528 10.1371/journal.pntd.0007843 PMC6957141

[4] Hashemi SA Badirzadeh A Sabzevari S First case report of atypical disseminated cutaneous leishmaniasis in an opium abuser in Iran.Rev Inst Med Trop Sao Paulo. 2018;60: e5. 29451599 10.1590/S1678-9946201860005 PMC5813668

[5] Arzamani K Visceral leishmaniasis in North Khorasan Province, north east of Iran. Int J Infect Dis. 2012;1 6:e340–e1.

[6] Badirzadeh A Mohebali M Sabzevari S Case report: First coinfection report of mixed *Leishmania infantum*/*Leishmania major* and human immunodeficiency virus–acquired immune deficiency syndrome: report of a case of disseminated cutaneous leishmaniasis in Iran. Am J Trop Med Hyg. 2018;98(1): 122–5. 29165208 10.4269/ajtmh.17-0490 PMC5928724

[7] Mirahmadi H Mansouri Nia M Ebrahimzadeh A Genotyping determination of Acanthamoeba strains: an original study and a systematic review in Iran. J Water Health. 2019;17:717–727. 31638023 10.2166/wh.2019.048

[8] Shafiei R Ghatee MA Jafarzadeh F Genotyping and phylogenetic analysis of unusually located hydatid cysts isolated from humans in north-east Iran. J Helminthol.2019;94:e64. 31331413 10.1017/S0022149X19000579

[9] Sarkari B Parhoode M Khabisi SA Genetic diversity of *Fasciola* spp. isolates from northern part of Iran: comparison with southwestern isolates. J Parasit Dis.2017;41:768–772. 28848276 10.1007/s12639-017-0886-6 PMC5555931

[10] Sarkari B Lari M Shafiei R A comparative seroprevalence study of toxocariasis in hypereosinophilic and apparently healthy individuals. Arch Pediatr Infect Dis. 2015;3:e17911.

[11] Badirzadeh A Mohebali M Asadgol Z The burden of leishmaniasis in Iran, acquired from the global burden of disease during 1990–2010. Asian Pacific J Trop Dis. 2017;7(9):513–518.

[12] Edrissian Gh H Nadim A Alborzi AV Visceral leishmaniasis: the Iranian experiences. Arch Iranian Med. 1998;1:22–6.

[13] Sharifi I Aflatoonian MR Parizi MHD Visceral Leishmaniasis in Southeastern Iran: A Narrative Review. Iran J Parasitol. 2017;12(1):1–11. 28761456 PMC5522684

[14] Mohebali M Arzamani K Zarei Z Canine visceral leishmaniasis in Wild Canines (Fox, Jackal, and Wolf) in Northeastern Iran using parasitological, serological, and molecular methods. J Arthropod Borne Dis. 2016;10(4):538–545. 28032106 PMC5186744

[15] Varma N Naseem S Hematologic Changes in Visceral Leishmaniasis/Kala Azar. Indian J Hematol Blood Transfus. 2010;26(3):78–82. 21886387 10.1007/s12288-010-0027-1 PMC3002089

[16] Karimi A Alborzi A Amanati A Visceral Leishmaniasis: An Update and Literature Review. Arch Pediatr Infect Dis. 2016;4(3):e31612

[17] Sampaio MJ Cavalcanti NV Alves JG Risk factors for death in children with visceral leishmaniasis. PLoS Negl Trop Dis. 2010;4(11):e877. 21072238 10.1371/journal.pntd.0000877 PMC2970542

[18] Sarkari B Rezaei Z Mohebali M Immunodiagnosis of Visceral Leishmaniasis: Current Status and Challenges: A Review Article. Iran J Parasitol. 2018;13(3):331–341. 30483323 PMC6243177

[19] Mohebali M Visceral leishmaniasis in Iran: review of the epidemiological and clinical features. Iran J Parasitol. 2013;8(3):348–58. 24454426 PMC3887234

[20] Rahi AA Ali MA Keshavarz Valian H Seroepidemiological studies of visceral leishmaniasis in Iraq. Sch J App Med Sci, 2013;1(6):985–989.

[21] Al-Hamash SM Study of visceral leishmaniasis (kala–azar) in children of Iraq. Mustansiriya Med J. 2012;1 1:15–9.

[22] Al-Orainey IO Gasim IY Singh LM Visceral leishmaniasis in Gizan, Saudi Arabia. Ann Saudi med. 1994;14(5):396–8. 17586953 10.5144/0256-4947.1994.396

[23] Chappuis F Sundar S Hailu A Visceral leishmaniasis: what are the needs for diagnosis, treatment and control? Nat Rev Microbiol. 2007;5(11):873–82. 17938629 10.1038/nrmicro1748

[24] Sarkari B Naraki T Ghatee MA Visceral Leishmaniasis in Southwestern Iran: A Retrospective Clinico-Hematological Analysis of 380 Consecutive Hospitalized Cases (1999–2014). PloS One. 2016;11(3):e0150406. 26942443 10.1371/journal.pone.0150406 PMC4778872

[25] Rodrigues V Cordeiro-da-Silva A Laforge M Regulation of immunity during visceral *Leishmania* infection. Parasit Vectors. 2016;9(1):118. 26932389 10.1186/s13071-016-1412-x PMC4774109

[26] Abdossamadi Z Seyed N Zahedifard F Human Neutrophil Peptide 1 as immunotherapeutic agent against *Leishmania* infected BALB/c mice. PLoS Negl Trop Dis. 2017;11(12): e0006123. 29253854 10.1371/journal.pntd.0006123 PMC5749894

[27] Srivastava P Dayama A Mehrotra S Diagnosis of visceral leishmaniasis.Trans R Soc Trop Med Hyg. 2011;105(1):1–6. 21074233 10.1016/j.trstmh.2010.09.006 PMC2999003

[28] Chulay JD Bryceson AD Quantitation of amastigotes of *Leishmania donovani* in smears of splenic aspirates from patients with visceral leishmaniasis.Am J Trop Med Hyg. 1983;32(3):475–9. 6859397 10.4269/ajtmh.1983.32.475

[29] Neki N Singh J Hematological changes in Visceral Leishmaniasis. Int J Curr Res Med Sci. 2017;3(6):36–40.

[30] Rai ME Muhammad Z Sarwar J Haematological findings in relation to clinical findings of visceral Leishmaniasis in Hazara Division. J Ayub Med Coll Abbottabad. 2008;20(3):40–3. 19610513

[31] Mirahmadi H Rezaee N Mehravaran A Detection of species and molecular typing of *Leishmania* in suspected patients by targeting cytochrome b gene in Zahedan, southeast of Iran. Vet World. 2018; 11(5):700–705. 29915511 10.14202/vetworld.2018.700-705 PMC5993770

[32] Marwaha N Sarode R Gupta R Clinico-hematological characteristics in patients with kala azar. A study from north-west India. Trop Geogr Med. 1991;43(4):357–62. 1812600

[33] Novotná M Havlíček J Smith AP *Toxoplasma* and reaction time: role of toxoplasmosis in the origin, preservation and geographical distribution of Rh blood group polymorphism. Parasitology. 2008;135(11):1253–61. 18752708 10.1017/S003118200800485X

[34] Flegr J Novotná M Lindová J Neurophysiological effect of the Rh factor. Protective role of the RhD molecule against *Toxoplasma*-induced impairment of reaction times in women. Neuro Endocrinol Lett. 2008;29(4):475–81. 18766148

[35] Flegr J Klose J Novotná M Increased incidence of traffic accidents in *Toxoplasma*-infected military drivers and protective effect RhD molecule revealed by a large-scale prospective cohort study. BMC Iinfect Dis. 2009;9:72. 10.1186/1471-2334-9-72 PMC2692860 19470165

